# Proteomics signatures associated with hip arthropathy in ankylosing spondylitis

**DOI:** 10.3389/fmed.2025.1556118

**Published:** 2025-05-14

**Authors:** Xianghui Wen, Linkai Fang, Zena Chen, Dong Liu, Shenghui Wen, Jinwei Li, Qiuxia Li, Qiujing Wei, Shuangyan Cao, Peng Zhang, Jieruo Gu

**Affiliations:** ^1^Shenzhen Longhua District Central Hospital, Shenzhen, China; ^2^Shenzhen Longhua Institute of Immunology Transformation, Shenzhen, China; ^3^Department of Rheumatology, The Third Affiliated Hospital of Sun Yat-sen University, Guangzhou, China; ^4^Department of Rheumatology, The Seventh Affiliated Hospital of Sun Yat-sen University, Shenzhen, China; ^5^Shenzhen Institutes of Advanced Technology, Chinese Academy of Sciences, Shenzhen, China

**Keywords:** ankylosing spondylitis, hip arthropathy, proteomics, hip joint tissues, biomarker

## Abstract

**Objective:**

Ankylosing spondylitis (AS) is a chronic inflammatory disease primarily affecting the axial skeleton and peripheral joints, with hip arthropathy representing a severe complication that critically impairs mobility. While persistent inflammation is a hallmark of AS, the molecular mechanisms driving hip involvement remain poorly characterized. This study aimed to identify and validate protein biomarkers associated with hip arthropathy progression in AS through integrated proteomic and functional analyses.

**Methods:**

Liquid chromatography-mass spectrometry (LC–MS/MS) was employed to screen for differentially abundant proteins (DAPs) in hip joint tissues from 30 AS patients and 14 non-AS controls. Bioinformatics methods were utilized to screen for and identify key DAPs.

**Results:**

A total of 2,050 proteins were relatively quantified, with 109 DAPs (34 upregulated and 75 downregulated) meeting the criteria of *p* < 0.05 and a fold change ≥1.5 or ≤0.67. Enriched GO terms represented by DAPs included the Wnt signaling pathway, MAPK cascade, and antigen processing and presentation of exogenous peptide antigen via MHC class I. The main Kyoto Encyclopedia of Genes and Genomes (KEGG) pathways included the PI3K-Akt signaling pathway, ribosome, metabolic pathways, and neutrophil extracellular trap formation. The protein–protein interaction (PPI) network identified ribosomal proteins (RPs), including RPS11, RPS24, RPL35, RPS3A, RPS6, RPS8, RPS14, and RPS7, as highly connected hub proteins. These RPs were significantly enriched in pathways associated with hip arthropathy pathogenesis, particularly osteoblast differentiation and T cell-mediated immune regulation.

**Conclusion:**

Based on proteomics approaches and bioinformatics analysis, this study discovered DAPs and signaling pathways associated with hip arthropathy in AS. It may provide potential as screening tools or therapeutic targets for AS, warranting further research for validation.

## 1 Introduction

Ankylosing spondylitis (AS) is a chronic inflammatory condition primarily characterized by persistent inflammation in the axial skeleton, and frequently accompanied by additional features such as peripheral arthritis (1), enthesitis, and dactylitis. Furthermore, it can manifest with extra-articular manifestations such as uveitis (2) and inflammatory bowel disease (3). This multifaceted disorder poses significant health risks, leads to a poor quality of life, and increases financial burdens on affected individuals.

Previous research has focused on elucidating the overarching pathogenesis of AS, spanning multiple disciplines such as genetics (4), gut microbiota (5), and drug sensitivity (6). Despite extensive investigations, the precise pathologic mechanism underlying AS remains largely unknown. It is widely believed to be immune-mediated and exhibits a strong genetic association with HLA-B27, a class I human leukocyte antigen (7, 8). However, research has also identified non-HLA genes involved in AS progression, along with specific inflammatory cytokines, such as tumor necrosis factor (TNF) (9), that contribute to the inflammatory process. Abnormal protein expression by multiple genes engages in diverse pathways across different diseased tissues, potentially serving as key drivers of AS (10). However, the intricate inflammatory processes involved in AS continue to pose significant challenges in understanding the full scope of its pathogenesis. Currently, there is no definitive, radical treatment available for AS, and the overall pathogenesis remains incompletely elucidated. In particular, there have been limited reports on the underlying mechanism of hip arthropathy in patients with AS. The complexity of AS necessitates ongoing research to unravel its underlying mechanisms and develop more effective therapeutic strategies.

Proteins serve as the ultimate products of gene expression and can elucidate the processes underlying disease manifestations more effectively and directly (11). Proteomics techniques are extensively utilized to analyze the relative abundances of proteins in two or more biological samples, thereby enabling the screening of biomarker proteins essential for diagnosis and prognosis (10). The proteomic analysis method based on liquid chromatography–tandem mass spectrometry (LC–MS/MS) offers robust capabilities and high precision in studying signal transduction (12). However, the challenges associated with acquiring tissue samples from AS patients have hindered proteomics research in this field. Notably, hip joint tissues obtained during hip replacement surgeries for AS-related hip arthropathy help address this limitation.

In this study, we employed MS-based proteomics approaches to analyze key proteins in the hip joint tissues of AS patients. This approach provides insights into AS hip arthropathy, enhancing our understanding of AS biology and identifying potential new therapeutic targets.

## 2 Materials and methods

### 2.1 Patients and samples collection

A total of 44 samples of hip joint tissues were collected during hip replacement surgery from patients in the Third Affiliated Hospital, Sun Yat-sen University. The cohort comprised an AS group (*n* = 30) and a non-AS control group (*n* = 14). The 1984 modified New York criteria for AS were fulfilled by all AS subjects (13). The non-AS group included patients with hip arthropathy or the fracture of the hip joint caused by trauma. The samples were obtained after obtaining informed consent from the patients. Liver and kidney functions in both groups were normal during the study period. These tissues collected during the operation were cleaned with phosphate buffered solution (PBS, Solarbio) and immediately stored in a −80°C freezer until used for the LC–MS/MS experiments. We collected clinical and laboratory results from the subjects, including age, gender, and blood chemistry. The study was approved by the ethics committee of the Third Affiliated Hospital of Sun Yat-sen University ([2022]02–007-02) and conducted in accordance with the Declaration of Helsinki.

### 2.2 Protein extraction and trypsin digestion

Hip joint tissue samples (20 mg) were homogenized in 400 μL of RIPA lysis buffer (Beyotime) supplemented with 1 × protease inhibitor cocktail (Promega) using a Servicebio tissue grinder (Servicebio Technologies, China). Pre-cooled 1.5 mL polycarbonate tubes containing 3 mm stainless steel grinding beads were used. Samples were flash-frozen in liquid nitrogen prior to homogenization to minimize protein degradation. The homogenization protocol consisted of 3 cycles of 90 s each at 30 Hz, with 30-s intervals on ice between cycles to prevent overheating. Following this, the homogenate was centrifuged at 12,000 × *g* for 20 min at 4°C to remove cell fragments. The supernatant was carefully collected and transferred to a new centrifuge tube. The protein concentration in the supernatant was determined using the BCA protein assay kit (Beyotime).

For each sample, 100 μg of protein was diluted to a final volume of 100 μL using RIPA buffer. Subsequently, 500 μL of pre-chilled acetone (J.T.Baker) was added to the protein solution, and the mixture was incubated at −20°C overnight to promote protein precipitation. The samples were centrifuged at 12,000 *g* for 20 min at 4°C to effectively precipitate the proteins with acetone. The precipitated proteins were then washed twice with 500 μL prechilled acetone to remove any residual contaminants. After washing, the supernatant was discarded, leaving the cleaned protein precipitate ready for further analysis.

The protein precipitate was re-dissolved using a 100 mM solution of triethylammonium bicarbonate (TEAB), which was prepared by diluting TEAB solution with 8 M urea to achieve the desired concentration. The protein solution was then reduced with TCEP (Sigma) for 60 min at 60°C, followed by alkylation with 25 mM iodoacetamide (IAA) for 30 min at room temperature, with the process conducted in the dark to prevent light-induced reactions. To ensure that the urea concentration in the protein samples was reduced to less than 1 M, 100 mM TEAB (without urea) was added. Subsequently, trypsin (Promega) was introduced to the protein samples at a trypsin:protein mass ratio of 1:100 for an initial digestion period of 4 h. This ratio was then adjusted to 1:50 for an overnight digestion. Trypsin digestion aims to fragment the proteins into smaller peptides, which are more conducive to downstream analytical processes.

The peptides obtained were subjected to desalting using Cleanert S C18 solid phase extraction columns (Agela-Phenomenex) following the manufacturer’s guidelines, before being dried in a vacuum centrifuge. The concentrated peptide pellets was reconstituted with 0.1% (v/v) aqueous formic acid for LC–MS/MS analysis.

### 2.3 LC–MS/MS analysis

Untargeted proteomics analyses were performed using a UHPLC U3000 system coupled with a Q Exactive HF-X mass spectrometer (Thermo Fisher Scientific). The UHPLC gradient was configured as follows: solvent A consisted of 0.1% formic acid in water, while solvent B was composed of 80% (v/v) acetonitrile. The gradient profile was 0–4.9 min at 5% B, 4.9–5 min with a linear increase from 5 to 12% B, 5–60 min with a linear increase from 12 to 26% B, 60–85 min with a linear increase from 26 to 42% B, 85–93 min: 90% B; 93–98 min: 90% B, 98.1 min: 5% B, and 98.1–110 min held steady at 5% B. Spray voltage was set to 2,100 V and capillary temperature was kept at 300°C. MS data were acquired using a Q Exactive HF-X Hybrid Quadrupole-Orbitrap mass spectrometer (Thermo Fisher Scientific), equipped with a heated ESI source, utilizing the full MS/dd-MS^2^ acquisition mode. For the acquisition of full MS data, the m/z scan range: 200–2000, with an automated gain control (AGC) target value of 3 × 10^6^, a maximum ion injection time of 30 ms, and a resolution of 60,000. For dd-MS/MS, the maximum IT was set to 30 ms at a resolution of 7,500, with an AGC target value of 2 × 10^5^, and the first fixed mass 100 m/z. The raw MS data were acquired on the Q Exactive HF-X Hybrid Quadrupole-Orbitrap using Xcalibur software (v4.3).

### 2.4 Data analysis

Raw MS data were processed using the Proteome Discoverer 2.4, with searches performed against the UniProt Human Reference Proteome database. Precursor and fragment ion mass tolerances were set to 10 ppm and 0.02 Da, respectively. For relative quantification, label-free quantification (LFQ) was applied using normalization based on total peptide amount to correct for batch effects. We used the ‘Wu Kong’ platform^1^ for the analysis of corresponding proteins. Missing values were imputed using the NAguideR algorithm with a k-nearest neighbors (*k* = 5) approach^2^ (14). Differentially abundant proteins (DAPs) were defined as those meeting a fold change threshold of ≥1.5 or ≤0.67 and a Benjamini-Hochberg adjusted *p*-value < 0.05. To thoroughly characterize the functional profiles of DAPs, we performed Gene Ontology (GO) enrichment analysis spanning the three primary categories of Biological Process (BP), Molecular Function (MF), and Cellular Component (CC), along with pathway enrichment investigations using the Kyoto Encyclopedia of Genes and Genomes (KEGG) and Reactome databases. All analytical procedures were implemented with the clusterProfiler R package (v4.0.5), with statistical significance determined by Benjamini–Hochberg false discovery rate (FDR) correction, maintaining a rigorous adjusted *p*-value threshold of <0.05.

The protein–protein interaction (PPI) network was constructed using STRING v12.0 (15), with parameters set as follows. The full STRING network was utilized; the interaction sources included experimental evidence, curated databases, co-expression, text mining, gene neighborhood, gene fusion, and co-occurrence, while interaction types encompassed both functional and physical associations. Edges in the network were defined by integrated evidence from multiple sources, with line colors corresponding to the specific type of interaction evidence. To ensure high-confidence interactions, a stringent interaction score threshold (>0.900, highest confidence level) was applied. Subsequently, the PPI network analysis data were imported into Cytoscape software version 3.10.0 for comprehensive network diagram analysis and image processing, allowing for a clearer understanding of their interactions and connections.

## 3 Results

### 3.1 Basic characteristics of the participants

In this study, a total of 44 hip joint tissues from 30 AS patients and 14 non-AS controls were collected to identify candidate biomarkers. The clinical and laboratory results are shown in Table 1. Key clinical parameters such as age, white blood cell count, lymphocyte count, neutrophil count, C-reactive protein (CRP), and erythrocyte sedimentation rate (ESR) were analyzed. For detailed data, refer to Table 1.

**Table 1:** The clinical characteristics and laboratory results of enrolled AS group and non-AS group.

Characteristics	AS (*n* = 30)	Non-AS (*n* = 14)	*p*
Male, *n* (%)	27 (90%)	12 (85.71%)	–
Age (years), mean ± SD	33.967 ± 4.635	36.357 ± 3.319	0.091
White blood cell count (×10^−9^/L), mean ± SD	7.338 ± 1.676	6.827 ± 2.337	0.412
Lymphocyte count (×10^−9^/L), mean ± SD	2.037 ± 0. 506	1.998 ± 0.636	0.826
Neutrophil count (×10^−9^/L), mean ± SD	4.141 ± 1.170	3.906 ± 1.695	0.594
CRP (mg/L), mean ± SD	12.028 ± 11.177	11.079 ± 8.436	0.779
ESR (mm/H), mean ± SD	37.067 ± 23.253	30.643 ± 24.406	0.405

AS, ankylosing spondylitis; SD, standard deviation; CRP, C-reactive protein; ESR, erythrocyte sedimentation rate.

### 3.2 Proteomics differentiates AS group and non-AS group

**Figure 1:**
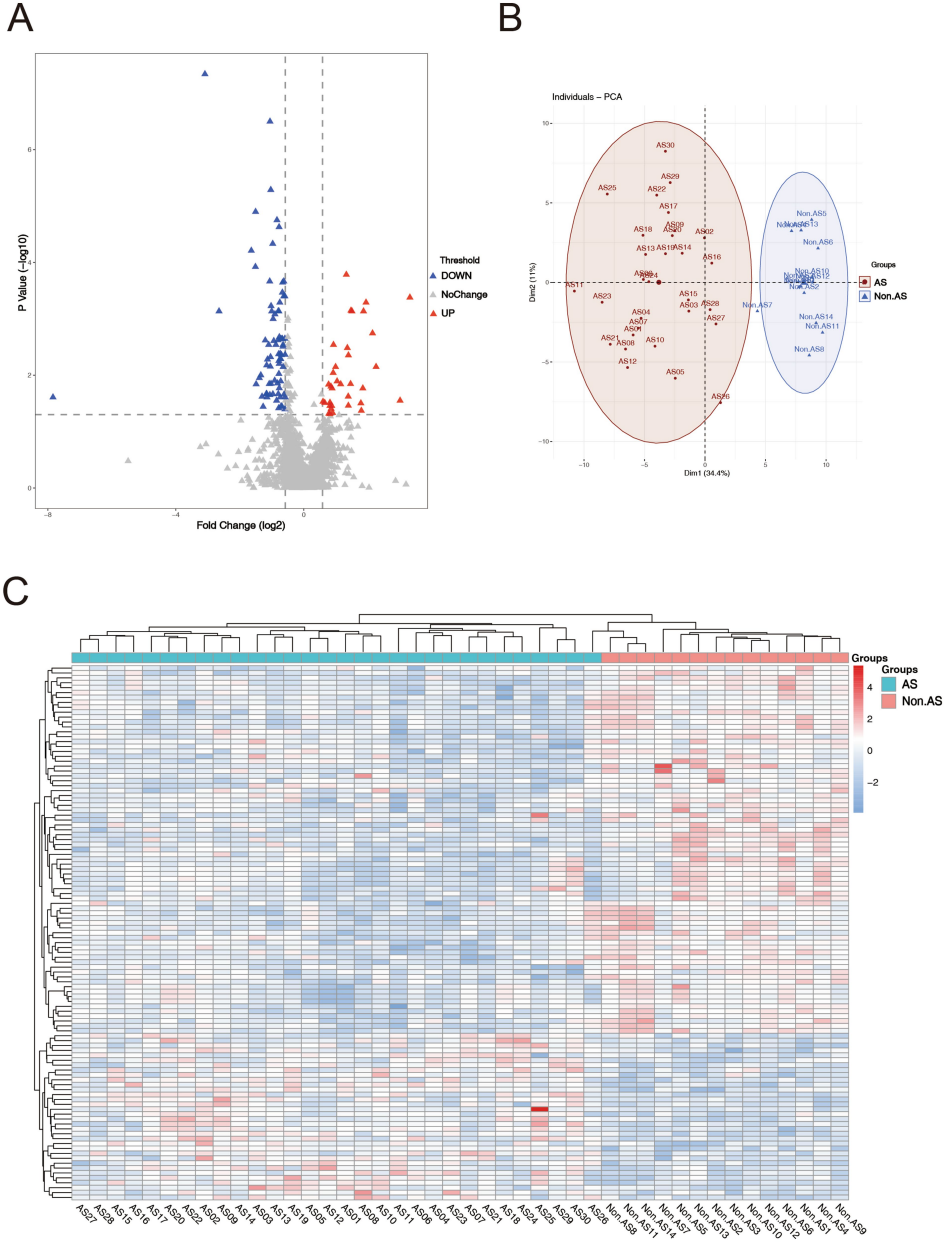
Enrichment analysis of DAPs. **(A)** Volcano plot of proteins, screening criteria of total proteome: fold change ≥1.5 or ≤0.67 and *p* < 0.05. Red dots represent upregulated proteins, and the blue dots represent downregulated proteins. Gray dots mean these proteins do not satisfy the screening criteria. **(B)** Principal component analysis (PCA) of AS patients and non-AS patients. **(C)** Heat map of the DAPs between AS and non-AS groups. Red areas denote upregulated proteins, and blue areas denote downregulated proteins.

LC–MS/MS analysis identified a total of 2,994 proteins, with 2,050 meeting quantification thresholds (Supplementary Table 1). Comparative analysis revealed 109 DAPs (34 upregulated, 75 downregulated) with *p* < 0.05 and fold change ≥1.5 or ≤0.67 (Supplementary Table 2). The volcano plot depicted significant differential expression patterns (Figure 1A). PCA analysis showed distinct clustering of AS and non-AS groups, characterized by proteins such as RPL18 and RPS6 (|loading score| > 0.8) (Figure 1B). These proteins were concurrently highlighted as significant DAPs in the volcano plot. The heatmap demonstrated consistent protein abundance patterns within the AS and non-AS groups, thereby reinforcing the reliability of differential expression results (Figure 1C).

### 3.3 GO functional enrichment analysis

**Figure 2:**
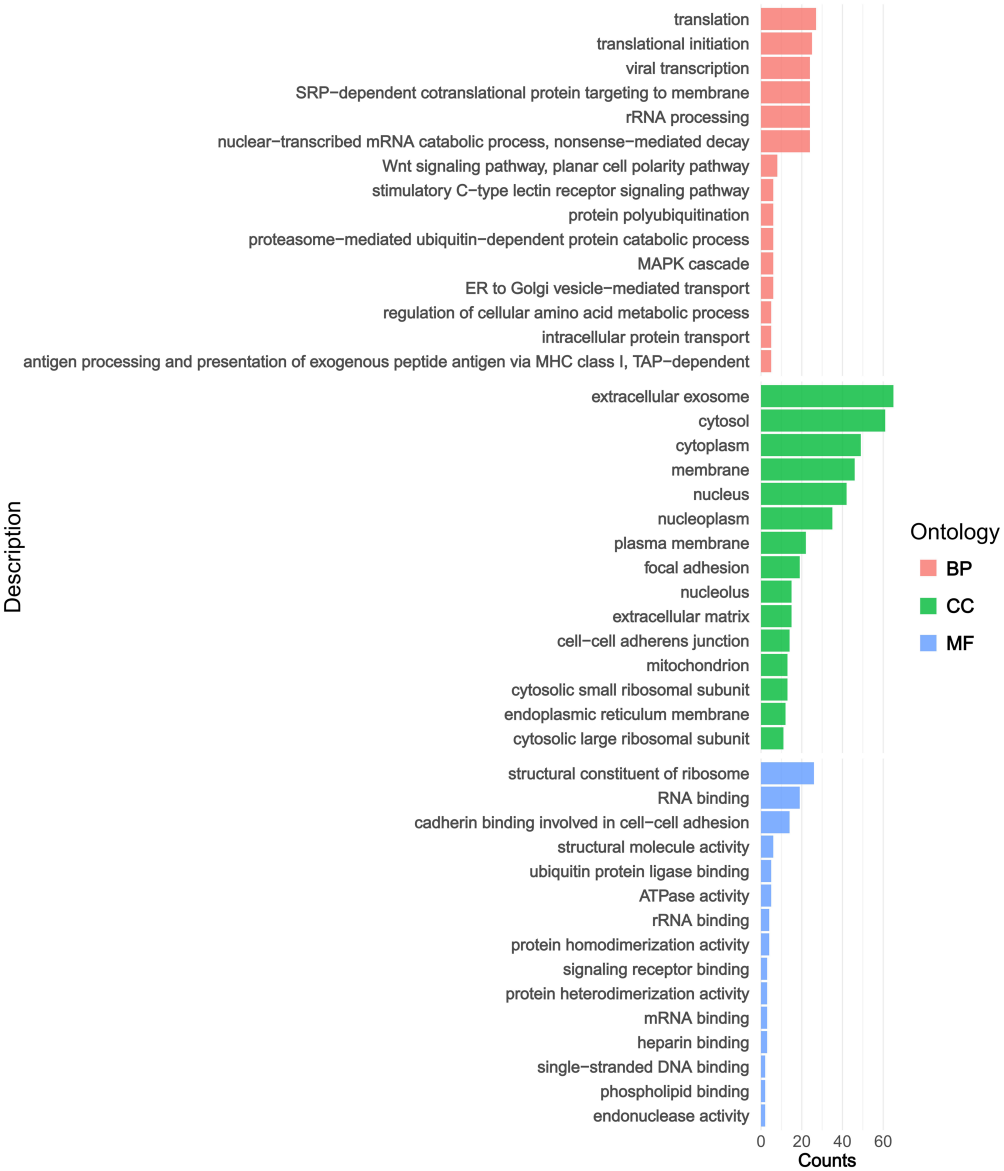
The pathway annotations derive from GO enrichment analyses. BP, biological process; MF, molecular function; CC, cellular component.

In terms of GO classifications, we identified distinct pathway groups for the DAPs, which were categorized into three main groups: BP, CC, and MF (Figure 2 and Supplementary Table 3). Within the BP category, GO enrichment analysis revealed that DAPs were significantly associated with processes including translation, translational initiation, viral transcription, and SRP-dependent co-translational protein targeting to membrane. In addition, our findings echoed those of previous studies on AS, highlighting key pathways such as the Wnt signaling pathway, the MAPK cascade, and the process of antigen processing and presentation of exogenous peptide antigen via MHC class I. In the CC category, proteins were predominantly associated with the extracellular exosome, cytosol, cytoplasm, and membrane. In the MF category, proteins were largely represented by structural constituents of ribosomes, RNA binding, cadherin binding represented by cell–cell adhesion and structural molecule activity.

### 3.4 KEGG pathway enrichment analysis

**Figure 3:**
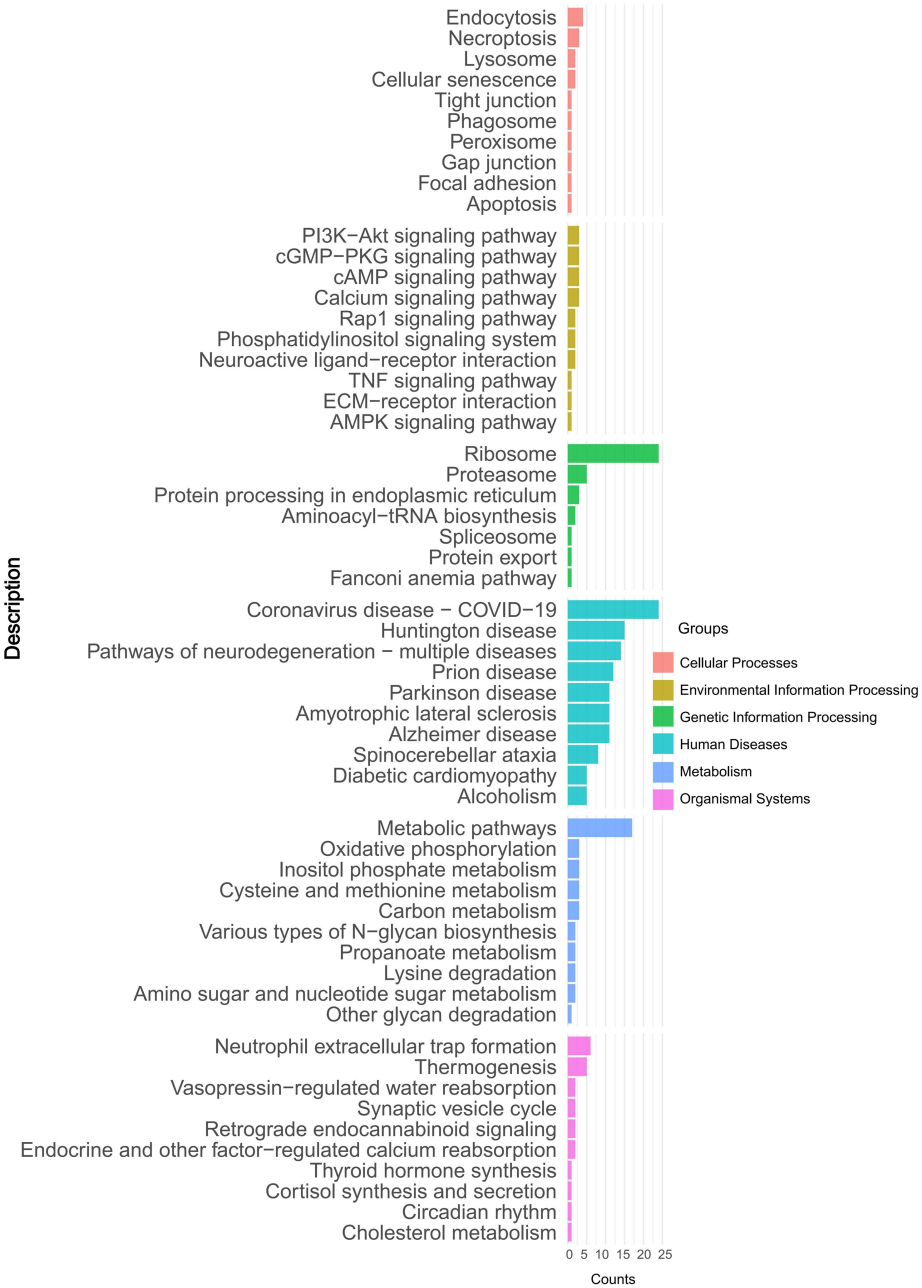
Kyoto Encyclopedia of Genes and Genomes (KEGG) pathway-based enrichment analysis of DAPs.

To identify the functionality of proteins that underwent significant changes, we performed a detailed KEGG pathway enrichment analysis. The results were visualized using a bar plot, depicting differential protein enrichment. Pathway analysis revealed six categories of the DAPs: Cellular Processes, Environmental Information Processing, Genetic Information Processing, Human Diseases, Metabolism, and Organismal Systems. Notably, DAPs were enriched in pathways such as the PI3K-Akt signaling pathway, Ribosome, Metabolic pathways, and Neutrophil extracellular trap formation. Among these, the Ribosome pathway showed the most significant enrichment (Figure 3 and Supplementary Table 4).

### 3.5 Reactome hierarchical enrichment analysis

**Figure 4:**
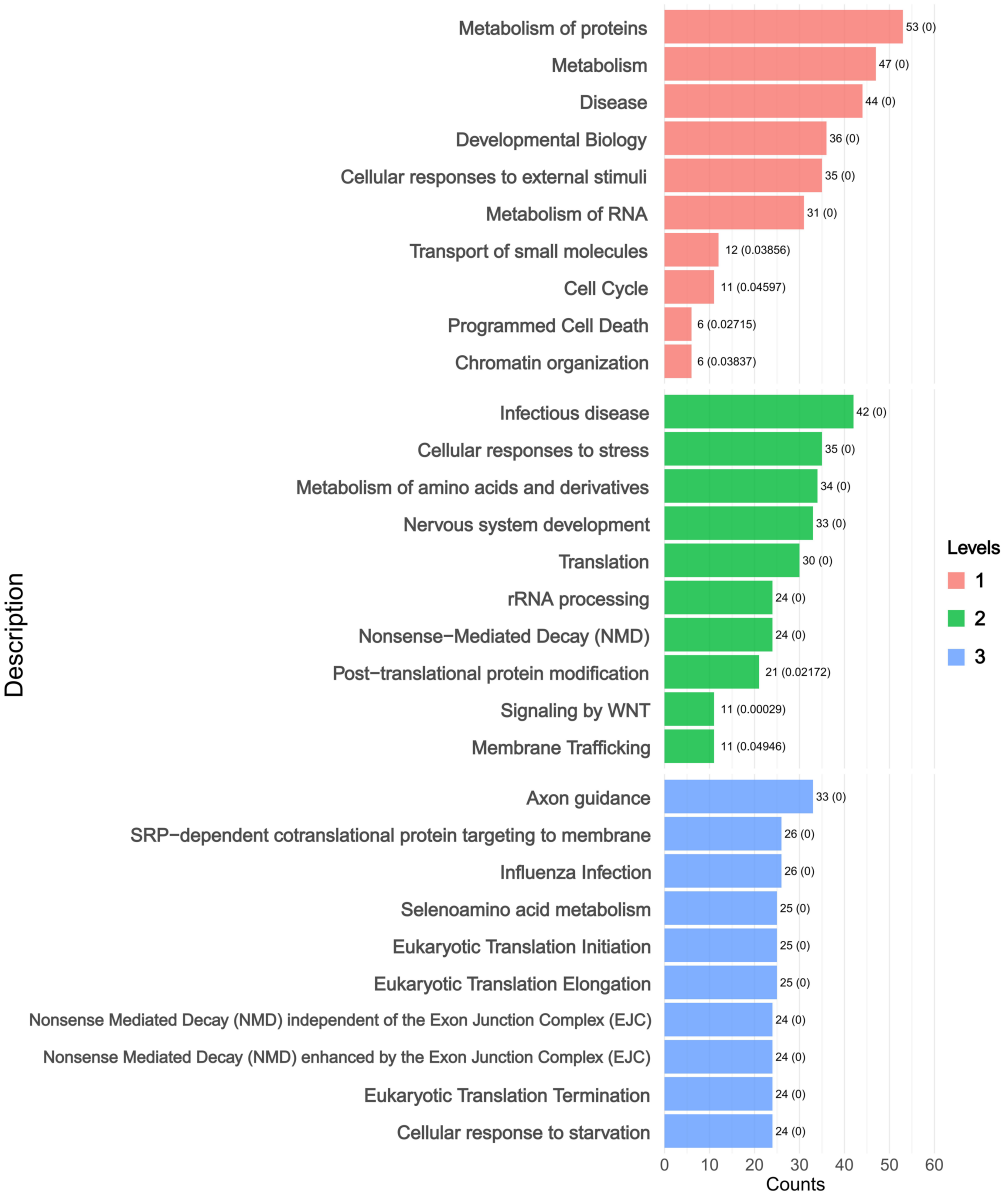
Reactome hierarchical enrichment analysis levels plot. (Level 1: broad categories; Level 2: intermediate subclasses; Level 3: specific pathways).

Hierarchical enrichment analysis was performed on DAPs (adjusted *p* < 0.05) using Reactome, which employs a hierarchical pathway classification system (Level 1: broad categories; Level 2: intermediate subclasses; Level 3: specific pathways). The pathway-centric analysis revealed significant enrichment of DAPs in key pathways including Protein Metabolism and Metabolic Disease Pathways. These pathways were sub-classified into Infectious Disease, Cellular Responses to Stress, and Amino Acid Metabolism, with further refinement identifying specific processes such as Axon Guidance and SRP-dependent Cotranslational Protein Targeting to Membrane (Figure 4 and Supplementary Table 5).

### 3.6 Protein–protein interaction network analysis

**Figure 5:**
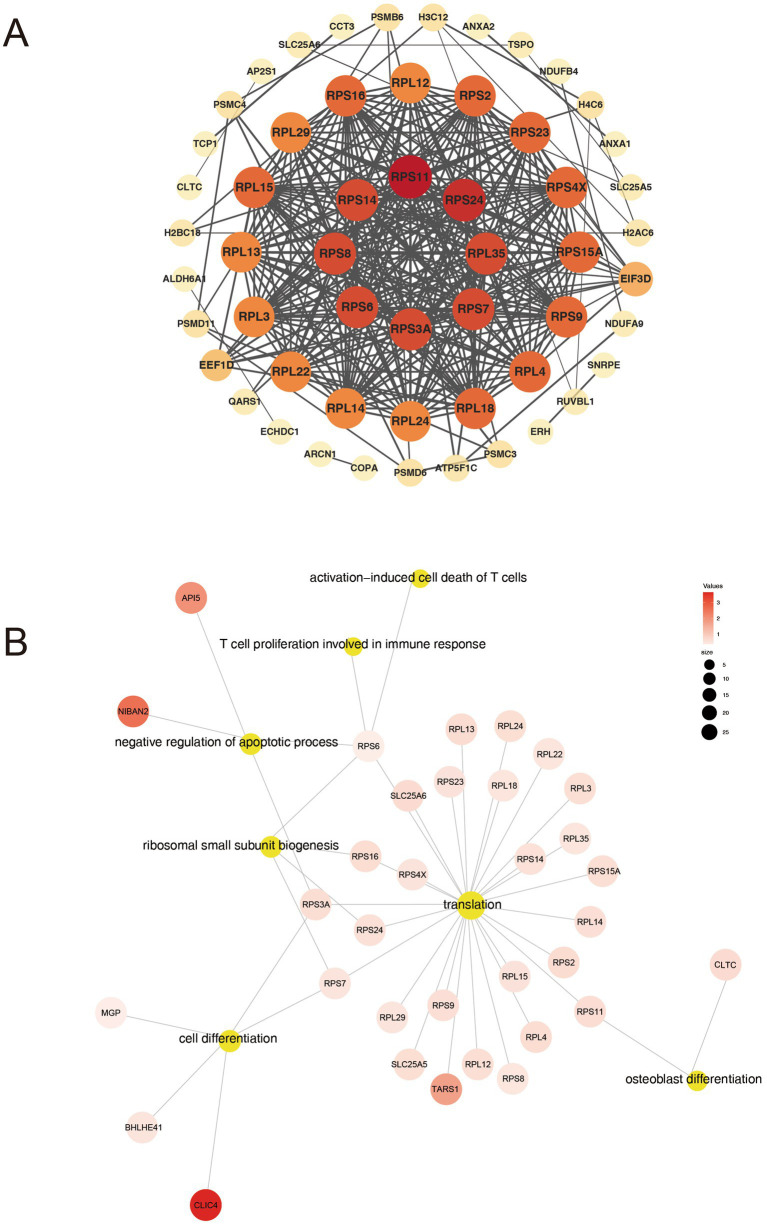
Analysis of PPI networks for DAPs. **(A)** Hub proteins in the PPI network. **(B)** GO biological processes associated with hub proteins.

To gain a deeper insight into the interplay among DAPs, we incorporated the 109 DAPs into a PPI network using the STRING platform (version 12.0). This integration aimed to assess interactions between the DAPs, and we retained only interactions with a STRING confidence score >0.900 (the database’s highest-confidence category). The resultant network comprised 108 nodes and 333 edges, where nodes with higher degrees of interaction were depicted in darker colors (Supplementary Figure 1). Using the CytoHubba plugin, we identified hub proteins with the highest interaction degrees in the PPI network, including RPS11 (degree = 27), RPS24 (degree = 26), RPL35 (degree = 25), RPS3A (degree = 25), RPS6 (degree = 25), RPS8 (degree = 25), RPS14 (degree = 25), and RPS7 (degree = 25) (Figure 5A). Among these, ribosomal proteins (RPs), including RPS11, RPS24, RPS6, and others, emerged as critical nodes exhibiting the highest connectivity, highlighting their central roles in shaping the network architecture. GO functional enrichment analysis of hub proteins within the PPI network, such as RPS11, RPS6 and RPS3A, identified biological processes closely associated with AS pathogenesis, including cell differentiation, osteoblast differentiation, and T cell-mediated immune responses (Figure 5B). Notably, RPs such as RPS11 or RPS6 emerged as highly connected hub nodes, enriched in pathways linked to osteogenic signaling and immune dysregulation.

## 4 Discussion

The AS is a persistent systemic inflammatory condition primarily impacting the sacroiliac joint and spine (16). Hip arthropathy is a common complication observed in AS patients. Chronic sacroiliitis associated with AS may lead to hip joint damage (17). However, the early diagnosis of AS is challenging due to its unclear pathogenesis and absence of a definitive diagnostic marker (18). In addition, the molecular mechanisms of hip arthropathy in AS have been rarely reported. The positional limitations of AS have largely confined studies on AS-related proteins. Most of the existing proteomic studies focus on serum (19), human synovial fluid (20), circulating plasma (21, 22) or plasma exosome proteomics (23). To promote our understanding of the pathogenesis underlying AS, it is imperative to explore additional potential proteins. LC–MS/MS-based proteomics is a powerful analytical technique that allows for the identification of proteins indicative of disease diagnosis and treatment (24). In this study, we conducted a comprehensive analysis of hip joint tissues from both AS and non-AS groups by integrating proteomics with bioinformatics. The aim of this study was to identify potential biomarkers for diagnosing AS-related hip arthropathy and monitoring disease progression.

In the present study, we discovered 109 DAPs in AS patients compared to non-AS groups. These 109 DAPs underwent GO enrichment analysis, with *P*. adj < 0.05. In terms of BP, our findings were consistent with previous studies on AS, highlighting key pathways such as the Wnt signaling pathway. Fibroblasts in the context of AS exhibit a robust osteogenic potential, which is characterized by a heightened capacity to form bone tissue. This osteogenic activity is accompanied by the activation of Wnt signaling pathways, a crucial regulatory mechanism represented by bone formation and differentiation (25). In addition, the results of the KEGG pathway enrichment analysis indicated that pathways linked to inflammation and ossification, specifically the PI3K-Akt and AMPK signaling pathways, were enriched in DAPs. Previous studies suggested that metformin exhibits a strong inhibitory effect on ossification and inflammation in AS fibroblasts by activating the PI3K-Akt and AMPK pathways (26). Furthermore, the research has shown that the PI3K-Akt signaling pathway is vital for controlling the proliferation, differentiation, and apoptosis of osteoblasts and osteoclasts. These processes are fundamental for maintaining bone homeostasis. This pathway is represented by bone formation and remodeling by affecting the activation state of downstream target proteins (27). Additionally, in the context of AS, the PI3K-Akt signaling pathway also exhibits a significant role that cannot be ignored in metabolism. AS patients tend to have low body fat due to high fatty acid oxidation. Insulin signaling was key in switching metabolism to fatty acids in AS. This shift involves increased gene expression regulating fatty acid oxidation, possibly mediated by PI3K (28). Furthermore, the KEGG analysis revealed that metabolic pathways exhibited the most prominent alterations within the category of metabolism, aligning with our previous research findings. Specifically, amino acid biosynthesis, glycolysis, fatty acid biosynthesis, and choline metabolism were the most prominently implicated pathways in AS (29). This finding was consistent with the enrichment results of metabolism within the Reactome pathway analysis. The PI3K-Akt signaling pathway, which influences both ossification and metabolism, is likely to play a significant role in the development of AS.

Antigen processing and presentation are crucial factors in the pathophysiology of AS (30). In our study, we found that the DAPs underwent GO enrichment analysis specifically in the process of antigen processing and presentation of exogenous peptide antigen via MHC class I, which was consistent with previous studies (31, 32).

The PPI network analysis identified RPs, particularly RPS11 and RPS6, as key DAPs in hip arthropathy tissues of AS patients. This finding aligns with the ribosome pathway’s prominence in our KEGG enrichment analysis and builds upon emerging evidence of ribosomal dysregulation in AS pathogenesis. Recent single-cell transcriptomic studies by Yarıcı and Karabekmez (33) revealed RPS11 as the most rewired interaction hub across immune cell subtypes in AS peripheral blood, whereas our tissue-level proteomics specifically implicate RPS11 in osteoblast differentiation pathways. Concurrently, RPS6 demonstrates dual functionality: it promotes mTOR-dependent osteoclastogenesis (34–36) and enhances T cell activation, directly linking translational regulation to immune-mediated joint damage. These mechanisms are consistent with cytokine-driven inflammation (such as TNF-*α*, IL-17) and Th17/Treg imbalance observed in AS (37).

The co-enrichment of RPs in osteogenic and immune pathways extends prior transcriptomic evidence of ribosomal dysregulation in AS. While Lari et al. (38) reported elevated RPL17 expression and pseudogene-mediated ribosomal alterations in peripheral blood mononuclear cells, the current study highlights distinct RP members (RPS11/RPS6) within affected joint tissues. This spatial divergence suggests RPs coordinate AS progression through two complementary axes: systemic immune dysregulation and localized osteogenic processes such as ectopic ossification (39). Although RPL35’s association with osteoarthritis and other skeletal disorders (40, 41) indicates broader musculoskeletal implications, our focused proteomic profiling reveals that RPs in AS hip tissues predominantly interact through pathways distinct from canonical translation. While altered RP abundance may reflect extraribosomal functions or ribosome biogenesis defects, these findings propose testable hypotheses: targeting RP-centric networks could modulate interconnected pathological mechanisms, offering novel strategies for managing AS arthropathy. Future studies should clarify whether ribosomal changes initiate pathology or emerge as secondary inflammatory biomarkers.

However, our research encountered several limitations that warrant careful consideration. First, our study employed a stringent fold-change threshold (≥1.5 or ≤0.67) to prioritize high-confidence DAPs; however, a relaxed threshold (≥1.2 or ≤0.83) might reveal subtler proteomic shifts. Given the limited sample size and inherent risk of false positives in exploratory proteomic studies, we focused on robustly altered proteins. Future studies with larger cohorts could adopt multi-threshold strategies to capture broader proteomic dynamics. Second, due to limited prior research on the association between AS and other RP family members beyond RPS11, RPS6, and RPL35, these proteins were not extensively discussed. Third, hip joint tissues from AS patients, particularly those with advanced arthropathy requiring replacement surgery, are rare and challenging to procure. Ethical and logistical constraints in tissue collection hindered molecular validation in this study. Fourth, while changes in RP abundance in AS may correlate with translation-associated processes, they do not directly confirm altered translational activity. Mechanistic validation through ribosome profiling, polysome analysis, and multi-omics integration is required to definitively characterize the functional implications of RP dysregulation in AS pathogenesis. Future work should correlate proteomic findings with longitudinal clinical data (e.g., radiographic staging, inflammatory markers) and explore strategies to address these limitations.

## 5 Conclusion

In conclusion, our study has explored the mechanisms of hip arthropathy in AS by combining proteomics and bioinformatics. We used LC–MS/MS to analyze samples from 30 AS patients and 14 non-AS patients, and identified 109 DAPs. Among these proteins, several key proteins, such as RPS11, RPS6, and RPL35, as well as others within the RP family, may play a central role in the development of hip arthropathy in AS. Their mechanisms could potentially be linked to osteoblast differentiation and T cell-mediated immune responses, particularly involving the PI3K-Akt signaling pathway that plays a central role in osteogenesis and metabolism. These proteins may serve as early screening tools or therapeutic targets for the disease. However, further research is needed to confirm these findings and understand their specific roles in the pathophysiology of AS.

## Data Availability

The raw proteomic data obtained by mass spectrometry (MS) in the current study is publicly available at the National Genomics Data Center (NGDC). It can be accessed via the following link: https://ngdc.cncb.ac.cn/bioproject/ with BioProject ID PRJCA039269 and accession number OMIX009961.

## Footnotes

^1^ https://www.omicsolution.com/wkomics/main/

^2^ https://www.omicsolution.com/wukong/NAguideR/

## Supplementary material

SUPPLEMENTARY FIGURE 1 Protein–protein interaction (PPI) network constructed using STRING v12.0 with a confidence threshold >0.900.

SUPPLEMENTARY TABLE 1 Normalized protein abundance matrix.

SUPPLEMENTARY TABLE 2 DAPs_statistics table.

SUPPLEMENTARY TABLE 3 Gene Ontology (GO) enrichment analysis tables.

SUPPLEMENTARY TABLE 4 Kyoto Encyclopedia of Genes and Genomes (KEGG) enrichment analysis tables.

SUPPLEMENTARY TABLE 5 Reactome enrichment analysis tables.

## References

[1] Kenyon M Gallagher P Dinneen B O’Shea F McManus R. Distinct clinical outcomes linked to peripheral arthritis and dactylitis in axial spondyloarthritis: findings from a retrospective irish cohort. Rheumatol Int. (2024) 44:2517–25. doi: 10.1007/s00296-024-05707-0, PMID: 39251445 PMC11424673

[2] Fernández-Torres J Zamudio-Cuevas Y Martínez-Flores K. Polymorphic variation of the defb1 gene might contribute to the development of ankylosing spondylitis: a preliminary study. Mol Biol Rep. (2024) 51:1051. doi: 10.1007/s11033-024-09985-6, PMID: 39395079

[3] Faggiani I Fanizza J D’Amico F Allocca M Zilli A Parigi TL. Extraintestinal manifestations in inflammatory bowel disease: from pathophysiology to treatment. Biomedicines. (2024) 12:1839. doi: 10.3390/biomedicines12081839, PMID: 39200303 PMC11351332

[4] Nancy Z Yan L Hui S Paul B Liye C. From the genetics of ankylosing spondylitis to new biology and drug target discovery. Front Immunol. (2021) 12:624632. doi: 10.3389/fimmu.2021.624632, PMID: 33679768 PMC7925991

[5] Jiang X Wang M Liu B Yang H Ren J Chen S. Gut microbiota and risk of ankylosing spondylitis. Clin Rheumatol. (2024) 43:3351–60. doi: 10.1007/s10067-024-07102-3, PMID: 39243281

[6] Chen J Wei C Huang S Wu S He R Chen T. Elucidating the causal nexus between antibody-mediated immunity and autoimmune diseases: insights from bidirectional mendelian randomization, gene expression profiling, and drug sensitivity analysis. Int Immunopharmacol. (2024) 142:113027. doi: 10.1016/j.intimp.2024.113027 39216119

[7] Im CJ Lee CY Beom JY Kim MG Yoon TR Park KS. Stricter correction of leg length discrepancy is required during total hip arthroplasty in patients with ankylosing spondylitis. BMC Musculoskelet Disord. (2023) 24:781. doi: 10.1186/s12891-023-06908-7, PMID: 37789293 PMC10546624

[8] Akassou A Bakri Y. Does hla-b27 status influence ankylosing spondylitis phenotype? Clin Med Insights Arthritis Musculoskelet Disord. (2018) 11:1179544117751627. doi: 10.1177/1179544117751627, PMID: 29343996 PMC5764146

[9] Yemula N Sheikh R. Gut microbiota in axial spondyloarthritis: genetics, medications and future treatments. ARP Rheumatol. (2024) 3:216–25. doi: 10.63032/WUII1201, PMID: 39243363

[10] Yu C Zhan X Liang T Chen L Zhang Z Jiang J. Mechanism of hip arthropathy in ankylosing spondylitis: abnormal myeloperoxidase and phagosome. Front Immunol. (2021) 12:572592. doi: 10.3389/fimmu.2021.572592, PMID: 34880852 PMC8647161

[11] Yin L Xu Y Mu J Leng Y Ma L Zheng Y. Cnksr2 interactome analysis indicates its association with the centrosome/microtubule system. Neural Regen Res. (2025) 20:2420–32. doi: 10.4103/NRR.NRR-D-23-01725, PMID: 39359098 PMC11759008

[12] Kanwal F Henry M Meleady P. Analytical tools for hcp detection, identification, and quantitation. Methods Mol Biol. (2025) 2853:235–48. doi: 10.1007/978-1-0716-4104-0_15, PMID: 39460924

[13] van der Linden S Valkenburg HA Cats A. Evaluation of diagnostic criteria for ankylosing spondylitis. A proposal for modification of the New York criteria. Arthritis Rheum. (1984) 27:361–8. doi: 10.1002/art.1780270401, PMID: 6231933

[14] Wang S Li W Hu L Cheng J Yang H Liu Y. Naguider: performing and prioritizing missing value imputations for consistent bottom-up proteomic analyses. Nucleic Acids Res. (2020) 48:e83. doi: 10.1093/nar/gkaa498, PMID: 32526036 PMC7641313

[15] von Mering C Huynen M Jaeggi D Schmidt S Bork P Snel B. String: a database of predicted functional associations between proteins. Nucleic Acids Res. (2003) 31:258–61. doi: 10.1093/nar/gkg034, PMID: 12519996 PMC165481

[16] Bi W Yang M Mao R. Unraveling shared diagnostic biomarkers of fibromyalgia in ankylosing spondylitis: evidence from comprehensive bioinformatic analysis and experimental validation. J Inflamm Res. (2024) 17:6395–413. doi: 10.2147/JIR.S474984, PMID: 39310900 PMC11415292

[17] Hu Z Li Y Hu L Ji X Wang L Li K. Cigarette smoking increases the prevalence of hip joint involvement in ankylosing spondylitis: a real-world case-control study. J Rheumatol. (2023) 50:901–6. doi: 10.3899/jrheum.220609, PMID: 36642427

[18] Diaconu AD Ceasovschih A Șorodoc V Pomîrleanu C Lionte C Șorodoc L. Practical significance of biomarkers in axial spondyloarthritis: updates on diagnosis, disease activity, and prognosis. Int J Mol Sci. (2022) 23:1561. doi: 10.3390/ijms231911561, PMID: 36232862 PMC9570274

[19] Hwang M Assassi S Zheng J Castillo J Chavez R Vanarsa K. Quantitative proteomic screening uncovers candidate diagnostic and monitoring serum biomarkers of ankylosing spondylitis. Arthritis Res Ther. (2023) 25:57. doi: 10.1186/s13075-023-03044-4, PMID: 37041650 PMC10088143

[20] Lee JH Jung JH Kim J Baek WK Rhee J Kim TH. Proteomic analysis of human synovial fluid reveals potential diagnostic biomarkers for ankylosing spondylitis. Clin Proteomics. (2020) 17:20. doi: 10.1186/s12014-020-09281-y, PMID: 32518534 PMC7269004

[21] Zhou Z Liu C Feng S Chen J Chen T Zhu J. Identification of novel protein biomarkers and therapeutic targets for ankylosing spondylitis using human circulating plasma proteomics and genome analysis. Anal Bioanal Chem. (2024) 416:6357–66. doi: 10.1007/s00216-024-05521-4, PMID: 39254691 PMC11541407

[22] Zhang Y Liu W Lai J Zeng H. Genetic associations in ankylosing spondylitis: circulating proteins as drug targets and biomarkers. Front Immunol. (2024) 15:1394438. doi: 10.3389/fimmu.2024.1394438, PMID: 38835753 PMC11148386

[23] Tavasolian F Lively S Pastrello C Tang M Lim M Pacheco A. Proteomic and genomic profiling of plasma exosomes from patients with ankylosing spondylitis. Ann Rheum Dis. (2023) 82:1429–43. doi: 10.1136/ard-2022-223791, PMID: 37532285

[24] Lai X Qi G. Using long columns to quantify over 9200 unique protein groups from brain tissue in a single injection on an orbitrap exploris 480 mass spectrometer. J Proteome. (2024) 308:105285. doi: 10.1016/j.jprot.2024.105285, PMID: 39159862

[25] Zeng Y Wang T Liu Y Luo T Li Q He Y. Wnt and smad signaling pathways synergistically regulated the osteogenic differentiation of fibroblasts in ankylosing spondylitis. Tissue Cell. (2022) 77:101852. doi: 10.1016/j.tice.2022.101852, PMID: 35753224

[26] Qin X Jiang T Liu S Tan J Wu H Zheng L. Effect of metformin on ossification and inflammation of fibroblasts in ankylosing spondylitis: An in vitro study. J Cell Biochem. (2018) 119:1074–82. doi: 10.1002/jcb.26275, PMID: 28696014

[27] Ma S Lin J Yang M Wang J Lu L Liang Y. Zhuangyao jianshen wan ameliorates senile osteoporosis in samp6 mice through modulation of the gcn5l1-mediated pi3k/akt/wnt signaling pathway. J Orthop Translat. (2024) 49:308–24. doi: 10.1016/j.jot.2024.08.009, PMID: 39568803 PMC11576941

[28] Xu WD Yang XY Li DH Zheng KD Qiu PC Zhang W. Up-regulation of fatty acid oxidation in the ligament as a contributing factor of ankylosing spondylitis: a comparative proteomic study. J Proteome. (2015) 113:57–72. doi: 10.1016/j.jprot.2014.09.014, PMID: 25281561

[29] Ou J Xiao M Huang Y Tu L Chen Z Cao S. Serum metabolomics signatures associated with ankylosing spondylitis and tnf inhibitor therapy. Front Immunol. (2021) 12:630791. doi: 10.3389/fimmu.2021.630791, PMID: 33679777 PMC7933516

[30] Wang G Kim TH Li Z Cortes A Kim K Bang SY. Mhc associations of ankylosing spondylitis in east asians are complex and involve non-hla-b27 hla contributions. Arthritis Res Ther. (2020) 22:74. doi: 10.1186/s13075-020-02148-5, PMID: 32272966 PMC7146985

[31] Yu Z Hong X Zhang X Zheng F Liu F Xu H. Global proteomic analyses reveals abnormal immune regulation in patients with new onset ankylosing spondylitis. Front Immunol. (2022) 13:838891. doi: 10.3389/fimmu.2022.838891, PMID: 35371008 PMC8967996

[32] Lu YP Zhang XL Zheng F Yun C Zhu C Cai W. Quantitative proteomic analyses to reveal the key features of proteins in new onset ankylosing spondylitis patients. ACS Omega. (2020) 5:20153–61. doi: 10.1021/acsomega.0c01776, PMID: 32832769 PMC7439379

[33] Yarıcı M Karabekmez M E. Deciphering metabolic pathways and protein-protein interaction networks in ankylosing spondylitis through single-cell RNA sequencing. (2024). [Epubh ahead of preprint]. doi: 10.1101/2024.10.21.619465

[34] Li J Liu X Cai C Zhang L An Z Guo Y. Plasma exosome-derived mir-455-5p targets rps6kb1 to regulate cartilage homeostasis in valgus-varus deformity (*Gallus gallus*). Poult Sci. (2024) 103:104169. doi: 10.1016/j.psj.2024.104169, PMID: 39244785 PMC11407033

[35] Yu FY Zheng K Wu YF Gao SW Weng QY Zhu C. Rapamycin exacerbates *staphylococcus aureus* pneumonia by inhibiting mtor-rps6 in macrophages. J Inflamm Res. (2023) 16:5715–28. doi: 10.2147/JIR.S434483, PMID: 38053607 PMC10695130

[36] Karg MM John L Refaian N Buettner C Rottmar T Sommer J. Midkine promotes metastasis and therapeutic resistance via mtor/rps6 in uveal melanoma. Mol Cancer Res. (2022) 20:1320–36. doi: 10.1158/1541-7786.MCR-20-0692, PMID: 35503453

[37] Chen T Ning S Zhu J Zhan X Zhou C Huang C. Exploring T cell and NK cell involvement in ankylosing spondylitis through single-cell sequencing. J Cell Mol Med. (2024) 28:e70206. doi: 10.1111/jcmm.70206, PMID: 39680481 PMC11648971

[38] Lari A Pourbadie HG Sharifi-Zarchi A Akhtari M Samimi LN Jamshidi A. Dysregulation of ribosome-related genes in ankylosing spondylitis: a systems biology approach and experimental method. BMC Musculoskelet Disord. (2021) 22:789. doi: 10.1186/s12891-021-04662-2, PMID: 34521416 PMC8442383

[39] Stovall R Kersey E Li J Baker R Anastasiou C Palmowski A. Incidence rate and factors associated with fractures among medicare beneficiaries with ankylosing spondylitis in the United States. Arthritis Care Res. (2024) 76:265–73. doi: 10.1002/acr.25219, PMID: 37605840 PMC10843294

[40] Zhu J Liu L Lin R Guo X Yin J Xie H. Rpl35 downregulated by mechanical overloading promotes chondrocyte senescence and osteoarthritis development via hedgehog-gli1 signaling. J Orthop Translat. (2024) 45:226–35. doi: 10.1016/j.jot.2024.01.003, PMID: 38596341 PMC11001632

[41] Li J Dong J Li M Zhu H Xin P. Potential mechanisms for predicting comorbidity between multiple myeloma and femoral head necrosis based on multiple bioinformatics. Comput Biol Chem. (2024) 113:108220. doi: 10.1016/j.compbiolchem.2024.108220, PMID: 39405776

