# Supplementary figures and images for: Proteomics signatures associated with hip arthropathy in ankylosing spondylitis
Source: Front Med (Lausanne). 2025 May 14;12:1556118. doi: 10.3389/fmed.2025.1556118 (PMC12116585; doi:10.3389/fmed.2025.1556118)

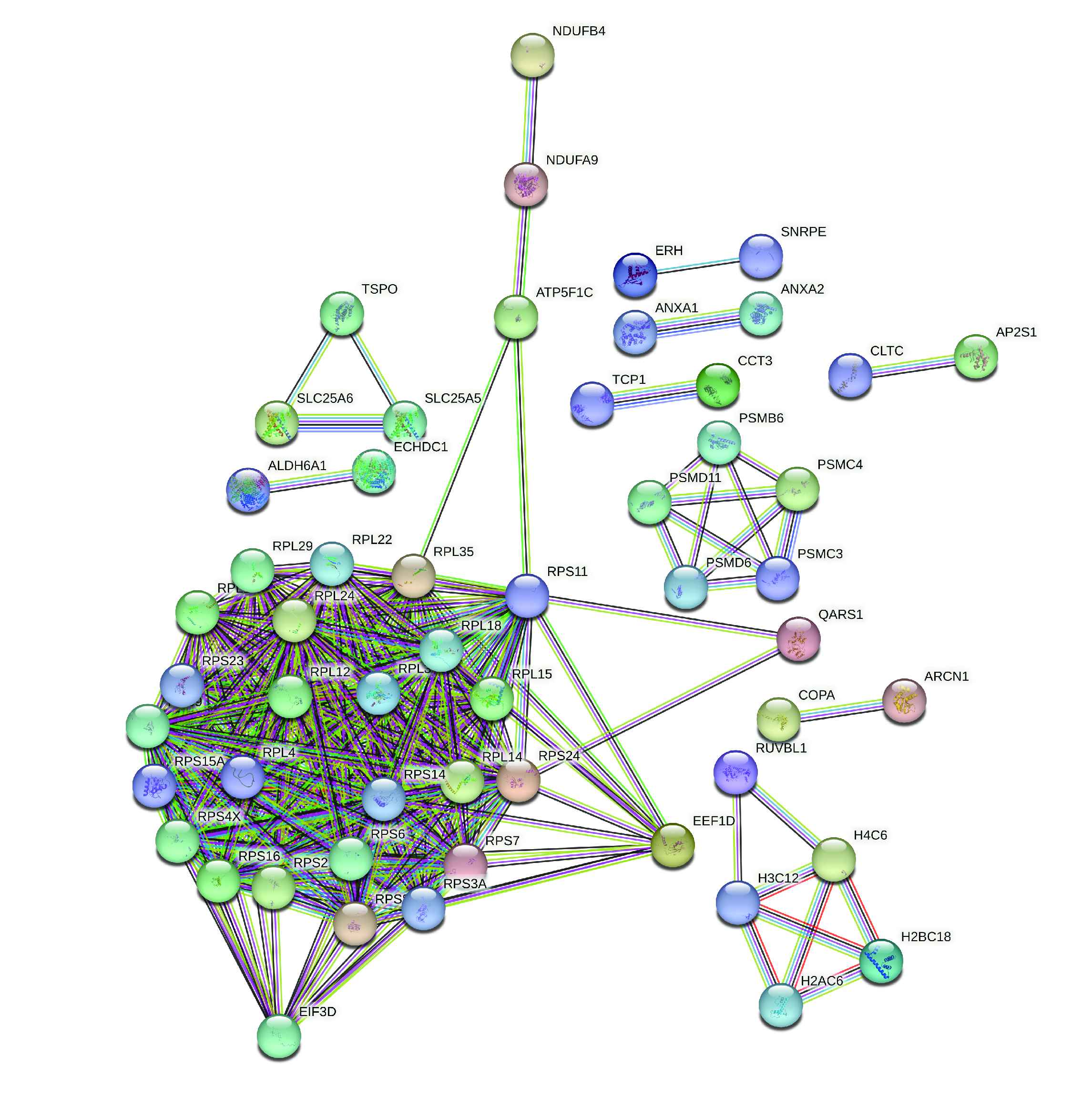

Supplement: SUPPLEMENTARY FIGURE 1 — Protein–protein interaction (PPI) network constructed using STRING v12.0 with a confidence threshold >0.900. [file Image_1.jpeg]
